# Effects of Common Fig (*Ficus carica* L.) and Its Extracts on Certain Cancer Types: Focusing on the Mechanism of Action

**DOI:** 10.3390/ijms27010056

**Published:** 2025-12-20

**Authors:** Elif Nisa Gökçen, Sevgi Gezici, Bence L. Raposa, Dávid Szép, Ferenc Budán, Duygu Ağagündüz

**Affiliations:** 1 Department of Nutrition and Dietetics, Faculty of Health Science, Kilis 7 Aralık University, 79000 Kilis, Turkey; elifnisa.pak@kilis.edu.tr; 2 Department of Medical Biology and Genetics, Faculty of Medicine, Gaziantep University, 27000 Gaziantep, Turkey; 3 Institute of Basics of Health Sciences, Midwifery and Health Visiting, Faculty of Health Science, University of Pẻcs, 7621 Pécs, Hungary; raposa.laszlo@pte.hu; 4 Institute of Physiology, Medical School, University of Pécs, 7624 Pécs, Hungary; szep.david@pte.hu; 5 Department of Nutrition and Dietetics, Faculty of Health Science, Gazi University, 06000 Ankara, Turkey

**Keywords:** *Ficus carica*, anticancer activity, bioactive compounds, plant extracts, phytochemicals

## Abstract

Cancer continues to be a global health problem due to high mortality rates and resistance to treatment. Since conventional chemotherapies cause serious side effects, interest in natural complementary therapies has increased. In this context, common fig (*Ficus carica* L.) (*F. carica*), which stands out with its rich phytochemical content, has been used in traditional medicine for a long time and attracts attention with its anticancer potential. The purpose of this review is to evaluate the biological effects of extracts obtained from different parts of the *F. carica* plant on cancer cells. Recent in vitro studies have shown that *F. carica* extracts suppress proliferation, induce apoptosis and reduce oxidative stress in various cancer cell lines. However, factors such as the plant part used, extraction method, dose and application time have caused differences in the results. In vivo studies are limited and there is no clinical study. Some studies report that high doses, especially latex, may cause toxic effects. *F. carica* extracts are promising against cancer. However, comprehensive in vivo and clinical studies with standardized extracts are needed to transfer this potential to clinical practice.

## 1. Introduction

Cancer is characterized by unregulated cell proliferation and remains the second leading cause of death worldwide. Globally, an estimated 20 million new cancer cases and 9.7 million deaths from cancer were reported in 2022 [1]. The cancer burden is projected to increase by approximately 77% by 2050, further straining healthcare systems, individuals, and communities [1,2]. One of the main goal in the field of cancer treatment is to formulate innovative approaches that specifically target malignant cells while sparing healthy tissues and reducing the development of resistance to chemotherapeutic compounds. Effective anticancer drugs are expected to exert their effects through mechanisms such as induction of apoptosis, inhibition of cell proliferation, promotion of cellular differentiation, suppression of DNA topoisomerase function, and modulation of intracellular signaling pathways. However, despite significant medical and technological advances, drugs used in traditional cancer treatments still face, in many cases, serious limitations, such as unacceptable toxicity, development of resistance, and the risk of targeting healthy cells and causing adverse effects [3,4]. Therefore, studies investigating the anticancer potential of naturally occurring compounds are gaining increasing importance. These limitations of conventional cancer therapies have intensified interest in exploring naturally occurring compounds as complementary or alternative strategies. Many plant-derived molecules interact with key oncogenic pathways—such as proliferation control, apoptosis induction, and oxidative stress regulation—suggesting that phytochemicals may help bridge current therapeutic gaps and support the development of safer, more targeted anticancer approaches [5,6]. In this context, plants that have been used in traditional medicine for a long time and have a rich pharmacological profile are attracting attention as alternative therapeutic agents. One of these plants, the Ficus genus, is an important group that stands out with its medicinal properties, and one of the most well-known species of this genus is the common fig (*Ficus carica L.)*, which is especially valued for its nutritious fruit [7,8]. *Ficus carica* L. (the common fig) has a long history of use in traditional medicine in the Mediterranean basin, the Middle East, North Africa, and parts of South Asia and East Africa. Different plant parts are used depending on tradition. The fruits and seeds are eaten for their nutritional and digestive benefits, while leaf decoctions are used for glycemic control and gastrointestinal complaints. The latex is applied topically for warts and other skin conditions. Ethnobotanical studies and pharmacological research document the use of the leaves, fruit, latex, bark, and roots for conditions such as digestive disorders, diabetes, skin conditions, infections, and inflammation. Examples include the traditional topical use of fig latex for verruca vulgaris and the use of fig leaf decoctions in experimental and clinical glycemic control studies [9,10,11]. This species, which naturally spreads from the intersection of Asia and Europe to Northern India, is commercially cultivated in many regions, especially in the Mediterranean countries, thanks to its high adaptability to hot climate conditions. However, its production is limited to certain regions due to its special pollination and drying requirements [12]. The fig is considered a quality fruit due to its high nutritional value. Beyond its nutritional benefits, *F. carica* serves as a rich source of polyphenols, flavonoids, and other bioactive compounds [13]. Minerals, vitamins, carbohydrates, phenolic compounds, organic acids and various sugars have been determined to be present in high amounts in dried fruits of *F. carica*. Fiber and polyphenol contents are remarkable in both fresh and dried fruits. Phytochemical analyses show that *F. carica* is a rich source not only of polyphenols and flavonoids, but also of many biologically active compounds such as β-amyrins, arabinose, β-carotene, β-sitosterols and xanthoxol, as well as various flavonoids and phenolic glycosides (including rutin and quercetin derivatives) [14,15]. Bioactive substances isolated from different parts of the plant include phenolic compounds, organic acids, phytosterols, coumarins, triterpenoids, aliphatic alcohols, and hydrocarbons. In addition to these groups, a number of volatile compounds and other secondary metabolites have been reported in *F. carica* cultivars. This richness of content is an important indicator supporting the pharmacological potential of the plant. As summarized in the review by Hajam and Saleem (2022) [16], several studies have reported that phenolic compounds, coumarins, flavonoids, and terpenoids isolated from *F. carica* may exert anticancer effects by inhibiting cancer cell proliferation, inducing apoptosis through mitochondrial pathways, and modulating oxidative stress. These conclusions are based on in vitro findings from human cancer cell lines, including breast, colorectal, and hepatocellular carcinoma models, in which isolated components such as psoralen, bergapten, and quercetin derivatives demonstrated cytotoxic or pro-apoptotic activity [16]. However, the current scientific evidence on these potential biological effects of *F. carica* is still limited. Comprehensive and systematic studies are needed to more clearly reveal the protective or therapeutic roles of different components of the plant on cancer development.

The aim of this review is to evaluate current scientific studies investigating the anticancer potential of extracts obtained from various parts of the *F. carica* plant. In line with the studies conducted in recent years, it has been reported that bioactive compounds obtained from different parts of the plant, such as fruit, leaf, latex, bark, and seed, have various biological effects on cancer cells through mechanisms such as inhibiting cell proliferation, inducing apoptosis, reducing oxidative stress and modulating tumor cell-specific signaling pathways [17,18,19]. In this context, in addition to variables such as plant part, extraction method, solvent, dose, and application time used in the review, the effects of the cancer cell lines used on the results were evaluated comparatively.

## 2. Bioactive Compounds of *F. Carica*

Phytochemical studies on *F. carica* have revealed that many biologically active compounds are present in various parts of the plant (latex, leaves, fruits, and roots). These compounds include phytosterols, flavonoids (including anthocyanins), organic acids, aliphatic alcohols and hydrocarbons. When examined in terms of organic acids, malic, citric, oxalic, shikimic and fumaric acids were detected, especially in the leaf extract, along with high amounts of quinic acid. In terms of flavonoid content, luteolin stands out as the main compound in the leaves, followed by quercetin and biochanin A. The antioxidant, anti-inflammatory and anticarcinogenic effects of these compounds have been reported in the literature. Phenolic compounds are found in all parts of the fruit; phenols such as caffeoylquinic acid isomers, quercetin derivatives, ferulic acid, psoralen, and bergapten were detected especially intensely in the leaves. Among phytosterols, β-sitosterol was found in the highest amount, while other sterols such as lupeol, α-amyrin, and betulol were also identified [14,16,20,21] (Table 1 and Table 2). In addition, the content of five different polyphenols (-epicatechin, chlorogenic acid, syringic acid, psoralen, and rutin) was determined in *F. carica* seeds [22].

## 3. Effects of *F. carica* on Cancer and Its Mechanism of Action

Studies investigating the anticancer effects of *F. carica* have revealed the potential therapeutic value of various extracts of this plant against different types of cancer. These studies, which were mainly conducted in in vitro and in vivo model systems, have revealed that extracts obtained from different parts of *F. carica,* such as fruit, leaves, latex and bark, exhibit various biological effects such as cytotoxicity, induction of apoptosis, cell cycle arrest, reduction in cell viability, suppression of migration and invasion [24,25,26,27]. It has been observed that factors such as the extraction methods used, type of solvent, dose intervals, and the exposure times are determinants of the biological effect. In addition, these studies report that the effects of *F. carica* mostly occur through mechanisms such as ROS production, disruption of mitochondrial membrane potential, caspase activation, p53 regulation and modulation of intracellular signaling pathways. However, some studies have also reported dose-dependent or cell line-specific ineffectiveness. Current evidence suggests that *F. carica* extracts exhibit significant cytotoxic and apoptotic effects, particularly on breast, liver, colon, and cervix cancer cell lines. Table 3 and Table 4 summarizes the findings from studies investigating the anticancer effects of *F. carica*.

### 3.1. Breast Cancer

The activity of *F. carica* against breast cancer has been investigated using extracts obtained from its latex, fruit, leaves and bark (Table 3).

A recent study has shown that exposure to varying concentrations (1/1500, 1/900, 1/600, 1/300, 1/180, 1/60, 1/36 and 1/12) of fig latex resulted in a significant decrease in the survival of breast cancer cells, exhibiting a dose-dependent response. The latex showed remarkable cytotoxic activity against MCF7 and MDA-MB-231 breast cancer models, especially at the highest concentration tested (1/12). Cell viability after treatment was observed as 20.63% and 16.66%, respectively, with half-maximal inhibitory concentrations (IC50) measured at 1/40 and 1/45 [34]. In a different study, researchers characterized the phenolic composition of the latex extract, identifying the presence of luteolin, rutin, quercetin, chlorogenic acid, and caffeic acid. Treatment of MCF-7 cells with this extract resulted in a significant decrease in cell viability. The strongest cytotoxic effect was observed at a concentration of 400 µg/mL, which reduced cell viability to 14 ± 2% (IC50: 134.60 ± 13.52 µg/mL). The researchers attributed this effect to the hydrophilic and hydrophobic interactions between phenolic compounds and proteins expressed by cancer cells. However, the study also reported that latex extract exhibited cytotoxic effects on healthy human skin fibroblasts cells (CCD-45 SK) [19]. Another study reported that *F. carica* latex exhibited antiproliferative and antimetastatic effects in MDA-MB-231 breast cancer cells. The treatment dose-dependently reduced cell proliferation and migration, induced apoptosis and led to morphological changes. Cytotoxic and genotoxic effects were confirmed by DNA damage analyses, and it was determined that these effects occurred at the molecular level by downregulation of AMPKα, ERK2, CREB, and GSK-3α/β signaling pathways [28].

**Figure 1:**
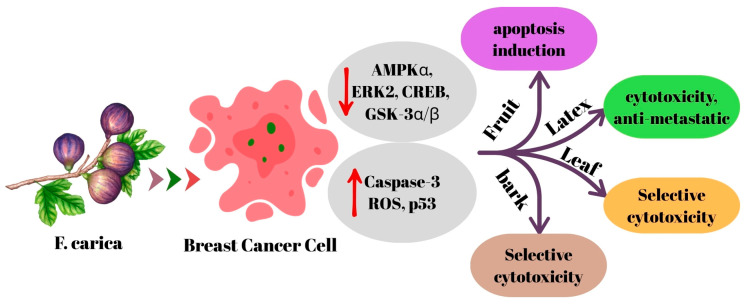
The mechanism of action of *F. carica* on breast cancer.

In a recent study, it was determined that *F. carica* leaf extract showed cytotoxic effects comparable to cisplatin in MCF-7, MDA-MB-231 and MDA-MB-436 breast cancer cells. This effect was reported to be related to caspase-3 activation by rutin, naringin and catechin compounds found in the extract. It was also reported that the extract showed lower toxicity against healthy peripheral blood mononuclear cells and was more selective against cancer cells [32]. A different study found that the leaf extract showed a significant inhibitory effect on cell viability in MCF-7 cells, while also exhibiting an increase in the percentage of cellular viability of peripheral blood mononuclear cells [25]. These differences from the findings reported by Yahiaoui et al. [19] are likely due to the distinct phytochemical compositions of latex and leaf extracts, which commonly lead to variations in biological activity rather than representing a true contradiction. In a study conducted by Farooq et al. [29], it was reported that nanoparticles obtained from *F. carica* leaf extract reduced cell viability in MDA-MB-231 breast cancer cells and increased the effectiveness of the chemotherapeutic drug integrated into these nanoparticles. Shiraishi et al. [31], reported that the leaf extract showed moderate cytotoxic effects in MCF-7 cells. In another study, it was observed that *F. carica* fruit extract promoted apoptosis in MCF-7 cells, but only had a mild cytotoxic effect [30]. In one study, 2-benzhydrylsulfinyl-N-hydroxyacetamide-Na compound found in *F. carica* fruit extract was identified and shown to trigger mitochondrial apoptosis in AMJ-13 breast cancer cells by increasing Bax, suppressing Bcl-2, as well as activating p53, caspase-8 and -9 via increasing ROS. The compound effectively inhibited cell proliferation and induced mitochondrial pathway-dependent apoptosis. Furthermore, no changes were observed in liver enzyme levels and lung and spleen tissues, and no significant toxicity was reported [24]. Finally, A recent study revealed that *F. carica* bark extract did not exhibit cytotoxic effects on healthy macrophage (RAW 264.7) and human skin fibroblast (CCD45-SK) cell lines, even at a high concentration of 400 µg/mL. However, the extract showed significant cytotoxic effect on MCF-7 breast cancer cells at lower concentration (IC50: 143.30 µg/mL), indicating its selective anticancer potential [33]. Ghandehari and Fatemi, in an in vivo experiment, demonstrated the anticancer effect of *F. carica* latex extract, decreasing tumor size and growth as well as inhibiting metastasis in breast tumor-bearing Wistar rats [47]. The mechanism of action of *F. carica* on breast cancer is summarized in Figure 1.

The evidence obtained indicates that *F. carica* has remarkable anticancer potential against breast cancer cells. Latex, leaf, fruit, and bark extracts have been reported to exhibit cytotoxic, antiproliferative, and apoptosis-inducing effects on different breast cancer cell lines. The latex modulates signaling pathways related to energy and proliferation, while leaf extracts stand out with caspase-3 activation and phytochemical selectivity. Compounds isolated from fruit extracts potently activate mitochondrial apoptosis, while the peel extract is notable for its low toxicity to healthy cells. Although the obtained findings are generally promising, it is understood that phytochemical differences between different types of extracts are decisive for the results. Therefore, before *F. carica* can be evaluated as a complementary agent in breast cancer treatment, extract standardization, toxicity profile, and mechanisms of action need to be investigated more comprehensively with advanced in vitro and in vivo studies.

### 3.2. Colon Cancer

The effects of *F. carica* against colon cancer have been evaluated in various studies with extracts from latex, fruit, leaves and bark of the plant.

A recent study investigated the impact of *F. carica* latex extracts on HT-29 colon cancer cells, revealing a dose- and time-dependent reduction in cell viability relative to the control group, both before and after digestion. Furthermore, both forms of the extract inhibited colony formation in HT-29 cells, though the undigested extracts exhibited lower IC50 values compared to their digested counterparts. In this study, the digested and undigested forms of F. carica latex extracts were compared using an in vitro gastrointestinal digestion model based on the standardized INFOGEST protocol [49]. Digestion was initiated by subjecting the extracts to sequential oral, gastric, and intestinal phases [38]. A study was conducted on HC-116 colon cancer cells to evaluate the cytotoxic effects of latex extracts from three different varieties of *F. carica*. The results indicated that the Aberkane and Aghanime varieties exhibited cytotoxic effects, while the El-bakour variety did not show any toxicity. Moreover, it was reported that all varieties exhibited elevated concentrations of cytotoxic activity against the healthy fibroblast cell line (CCD-45SK) [19]. Similarly, another study found that latex obtained from the Yellow Lop variety did not show cytotoxic effects at 10 μg/mL, but reduced HT-29 cell viability by 28.91% after 72 h at 20 μg/mL. Alternatively, latex from the Aydın Siyah variety at the same concentration caused 85.33% cell death. Furthermore, 40 μg/mL Aydın Black latex increased apoptosis by 10-fold, while Yellow Lop latex increased it by 2-fold, and this effect was shown to be related to caspase-3 and -7 activation [35]. The findings of these two studies reveal that *F. carica* variety is an important factor in terms of biological activity. The difference in activity between varieties may be due to differences in their phytochemical compositions. A recent study reported that nanofibers loaded with *F. carica* latex showed lower toxicity compared to 5-fluorouracil on normal cells (WI-38) and had significant cytotoxic effects on colon cancer cells (Caco-2) (IC50: 23.96 μg/mL). Molecular analysis revealed upregulation of *BCL-2* and downregulation of *P53* and *TNF*, indicating that this treatment could potentially weaken tumor suppressive mechanisms. However, increased *P21* gene expression suggests that the antiproliferative effect may be supported by cell cycle arrest [17].

**Figure 2:**
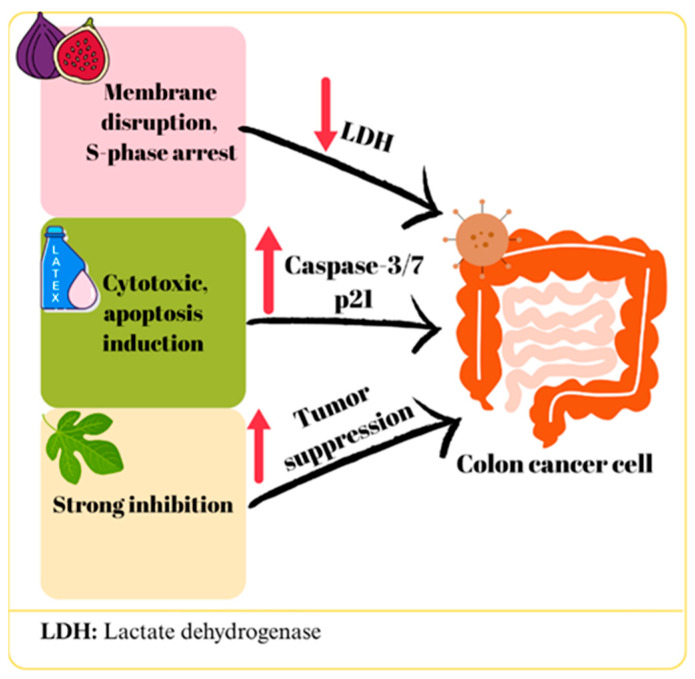
The mechanism of action of *F. carica* on colon cancer.

In a study using the Caco-2 cell line, a significant inhibition of cell proliferation was observed when *F. carica* leaf extract was applied in the concentration range of 5000–156 μg/mL [25]. However, in another study using a similar cell line, no significant cytotoxic effect was detected at an extract concentration of 8 mg/mL [31]. Despite using a comparable cell line and applying concentrations within a similar range, the discrepancies in results may stem from variations in the specific *F. carica* cultivar used, differences in incubation periods, or unavoidable phytochemical degradation occurring between leaf collection and extract preparation. Abdou et al. [36] evaluated the in vitro and in vivo cytotoxic effects of bergapten isolated from *F. carica* leaves on C-26 colon cancer cells by loading it into cubosomes. Bergapten-loaded cubosomes exhibited stronger cytotoxicity than free bergapten (IC50 values of 0.015 ± 0.001 μM and 0.062 ± 0.003 μM, respectively, for 72 h incubation), while empty cubosomes did not show toxicity. Additionally, significant reductions in tumor volume and weight were observed in mice treated with bergapten cubosomes in in vivo experiments [36]. A significant cytotoxic effect was observed in HT-29 cells treated with *F. carica* fruit extract, depending on the dose and time. The increase in lactate dehydrogenase (LDH) levels in the culture medium suggested that this cytotoxic effect may be due to the disruption of cell membrane integrity. Furthermore, the researchers reported that the extract arrested HT-29 cells in S phase (*p* < 0.007) and that apoptosis rates increased 12-fold in the early phase and 1.5-fold in total after treatment [37]. In a study in which HT-116 cell lines were treated with *F. carica* bark extract, only a negligible cytotoxic effect was observed even at the highest concentration (400 μg/mL), but this varied among different cultivars [33]. According to Sharma et al. the morin flavonoid content of *F. carica* inhibited the 1,2-dimethylhydrazine-induced colon cancer in Wistar rats by downregulating the inflammatory and cell proliferation-stimulating NF-κB pathway, including interleukin 6 (IL-6), TNF-α, prostaglandin (PGE-2), and cyclooxygenase 2 (COX-2). Furthermore, it increased the BAX/BCL2 ratio, favoring the apoptotic pathway [48]. Hence, morin downregulates both Pumilio-1 (PUM1) and CD133, leading to inhibition of Cancer Stem Cells (CSCs) that may remain after colon cancer treatment [50]. The potential effects of *F. carica* on colon cancer are summarized in Figure 2.

Various studies on colon cancer cells have demonstrated that extracts from different parts of *F. carica* exhibit significant effects, which are dependent on both dose and plant variety. In particular, it appears that the digestive process can weaken the biological activity of extracts, while differences in plant variety can significantly alter toxicity levels. Furthermore, the use of nanotechnological formulations such as cubosomes and nanofibers has been reported to increase the efficacy of extracts while potentially reducing toxicity to healthy cells. However, the inconsistencies in the levels of efficacy observed in different studies are due to differences in the *F. carica* varieties used, extract preparation methods, and experimental conditions. This suggests that the potential of *F. carica* in the treatment of colon cancer depends on the development of standardized extract preparation and dosing protocols. Overall, the data support the existence of anticancer effects of the plant, but its efficacy needs to be optimized and its mechanisms investigated in more detail.

### 3.3. Liver Cancer

The therapeutic capacity of *F. carica* against liver cancer has been addressed in studies evaluating extracts from various tissue parts, including latex, fruit, leaves, and bark.

**Figure 3:**
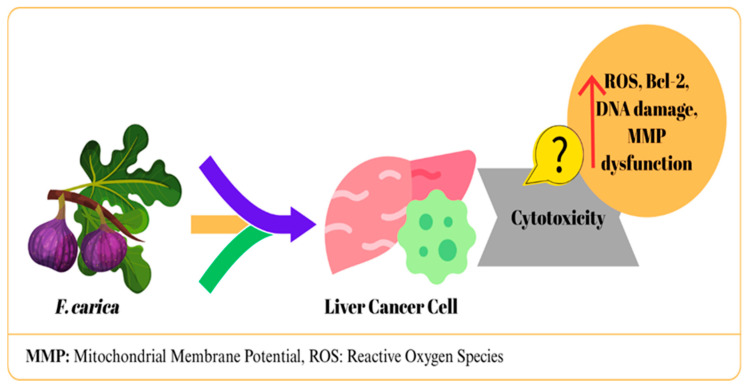
The mechanism of action of *F. carica* on liver cancer.

In a study, *F. carica* latex-loaded nanofiber was determined to have cytotoxic effects on liver cancer cells (HepG2), and the IC50 value was determined as 23.97 μg/mL [17]. Similarly, in HepG2 hepatocellular carcinoma cells, exposure to the highest tested concentration (100 µg/mL) of latex from the three *F. carica* cultivars (Aberkanei, Aghanime, and El-Bakour) resulted in a marked decrease in cell viability, with the highest IC50 value reported as 19.40 ± 2.69 µg/mL [19]. In another study, *F. carica* latex was tested with four different solvents in HepG2 cells. The methanol fraction exhibited no cytotoxic activity and promoted cell growth, while the other fractions showed toxic effects. The strongest effect was observed in the chloroform fraction with an IC50 value of 0.2 ± 0.02 mg/mL and was reported to induce apoptosis. This effect was attributed to lupeol acetate and lupeol palmitate identified in the fraction [26]. Mustafa et al. [39] reported that *F. carica* leaf extract produced dose-dependent cytotoxic effects on HepG2 cells (IC50: 0.081 mg/mL), whereas it did not show significant toxicity up to 2 mg/mL in healthy HUVEC cells. While significant morphological deteriorations were observed in treated HepG2 cells, HUVECs maintained their normal appearance. These differences suggested that the extract exhibited selective apoptotic effects. The researchers stated that this effect was associated with increased ROS production, DNA damage, disruption of mitochondrial membrane potential, suppression of Bcl-2, and downregulation of CDK-1, CDK-5, and CDK-9 [39]. Similarly, Abdel-Rahman et al. [25] reported that leaf extract showed a significant effect in HepG cells with a 66.9% inhibition rate [25]. A study reported that treatment of HepG2 cells with *F. carica* dried fruit extract provided moderate cytotoxic effects [30]. In one study, the anticancer effects of *F. carica* fruit essential oils extracted using a hot distillation procedure with water (FCFW) and hexane (FCFH) were investigated in HepG2 cells. It was determined that both extracts showed moderate cytotoxicity; FCFW arrested the cells in S phase, while FCFH did not affect the cell cycle. In the apoptosis analysis, FCFH increased early apoptosis (43.1%), while FCFW increased late apoptosis (30.2%) [40]. In addition, it was observed that both extracts reduced ROS production, which contradicts what was reported by Mustafa et al. (2021) [39]. This discrepancy indicates differences in the anticancer mechanisms of fruit and leaf extracts and suggests that ROS reduction may be a different pathway from classical chemotherapeutic mechanisms [40]. Finally, *F. carica* wood bark extract showed a weak cytotoxic effect against HepG-2 cells (IC50: ≥100 μg/mL) [33]. The potential effects of *F. carica* on liver cancer are summarized in Figure 3.

In general, different tissue extracts of *F. carica* show anticancer potential in HepG2 cells. Notably, noteworthy that nanofiber formulations increase efficacy while reducing toxicity to healthy cells. The findings vary depending on the tissue used, extraction method, and test conditions. Therefore, standardization of extracts, isolation of active components, and more comprehensive studies at the mechanistic level are required to fully demonstrate the therapeutic potential of F. carica against liver cancer.

### 3.4. Cervical Cancer

The effects of *F. carica* on cervical cancer were evaluated through studies performed with latex and fruit-derived extracts of the plant.

**Figure 4:**
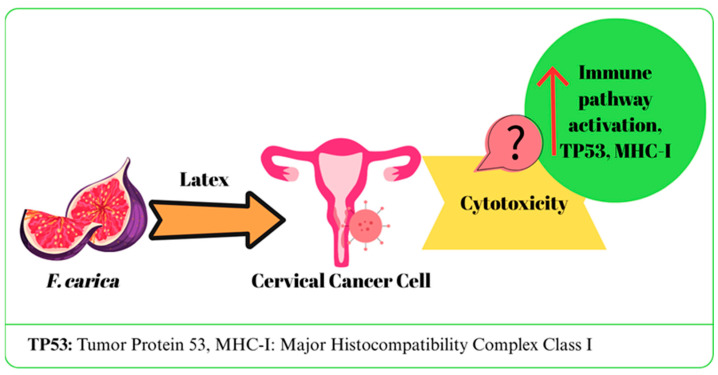
The mechanism of action of *F. carica* on cervical cancer.

A study investigated the anticancer activity of digested (in vitro) and undigested *F. carica* latex (white and black varieties) in the HeLa cell line. Although in vitro gastrointestinal digestion was observed to cause a decrease in anticancer properties, all calculated % viability values were reported to be lower compared to the control groups [38]. In two similar studies, the effects of *F. carica* latex on HPV16+ CaSki, HPV18+ HeLa, and HPV-negative C33A cervical cancer cell lines were evaluated. IC50 values were determined as 106 µg/mL for HeLa, 110 µg/mL for CaSki, and 108 µg/mL for C33A, and no toxicity was observed in healthy HCKT1 cells. Latex application led to upregulation of genes related to immune response, RNA processing and protein degradation, especially in HeLa and CaSki cells, while it activated apoptosis pathways in C33A cells. In addition, it was determined that MHC-I expression and TP53-mediated pathways increased, and the immune escape effect of E5 protein decreased. It has been reported that latex affects cell cycle and immune responses through regulatory molecules such as TAF1, CDK1/4, and MAPK1 [41,42]. In a different study, it was suggested that *F. carica* fruit extract did not exhibit any cytotoxic effect on HeLa cancer cells (IC50: >1000 µg/mL). Furthermore, when combined with olive oil, it diminished the cytotoxic effect of olive oil [27]. The potential effects of *F. carica* on cervical cancer are summarized in Figure 4.

Current studies on cervical cancer cells have shown that latex extract the latex extract has a unique activity profile, specifically targeting genetic and immune response mechanisms. In addition, its selective effect is supported by the fact that it does not cause toxicity in healthy cells. However, no cytotoxic effect was observed with the fruit extract. These findings show that the potential of *F. carica* varies depending on the part of the plant and the method of application. Further in vivo and clinical studies are needed to confirm the efficacy and clarify the mechanisms.

### 3.5. Other Types of Cancer

The fruit extract of *F. carica* has been the subject of investigation in the context of pancreatic, ovarian, gastric, and osteosarcoma cancer cell lines.

In one study, the extract (0.1, 0.5, 1.0 mg/mL for 24 h) exhibited low toxicity in Panc-1 and AsPC-1 pancreatic cancer cells, with no significant antiproliferative effect. While the extract slightly reduced cancer cell viability, it did not substantially impact cell proliferation [44]. Conversely, another study reported that *F. carica* fruit extract exhibited dose- and time-dependent cytotoxic effects on pancreatic cancer cells. The 600 µg/mL extract caused a 60% and 70% decrease in viability in Panc-1 and QGP-1 cells, respectively. Apoptosis was confirmed by increased PARP-1 activation, and cell migration and invasion were suppressed. The extract induced cell death by increasing ROS production, while simultaneously balancing this effect by stimulating autophagy. The decrease in mitochondrial membrane potential and the increase in lipid peroxidation suggested the potential for ferroptosis. In vivo experiments, oral administration of 200 mg/kg extract significantly suppressed tumor growth in mice with QGP-1-induced tumors; increased PARP-1 and LC3A/B expression indicated a collaborative role for apoptosis and autophagy. No toxicity was observed in normal pancreatic cells [43]. The observed variations in results between the two studies may be due to differences in extract composition, solvent choice, preparation techniques, cell models, administered concentrations, exposure duration, and overall experimental preparation. Notably, the extract’s impact on cancer cells seems influenced by both concentration and treatment duration.

Ali et al. [45] reported that the 2-(benzhydrylsulfinyl)-N-sec-butylacetamide compound isolated from *F. carica* fruit promoted macrophage activation in SKOV-3 ovarian cancer cells by increasing Fcγ receptor expression. When co-administered with trastuzumab, the compound enhanced phagocytic activity and exerted in vivo tumoricidal effects through the induction of reactive oxygen species (ROS) generation and mitochondrial activation. Similarly, 2-benzhydrylsulfinyl-N-hydroxyacetamide-Na compound derived from the same plant showed dose-dependent suppression of proliferation and induction of apoptosis in SKOV-3 cells. This effect was associated with downregulation of BCL-2, activation of pro-apoptotic factors, and cytochrome c release due to a decrease in mitochondrial membrane potential [24].

A recent study reported that *F. carica* fruit extract exhibited cytotoxic effects in gastric cancer cells (AGS) in a dose-dependent manner by both increasing ROS production and via apoptosis and DNA damage [18]. Another study reported that the fruit extract promoted cell death in osteosarcoma cancer cells (MG-63) in proportion to dose and time by disrupting the cell membrane structure, reducing mitochondrial activity and DNA damage, although no significant changes were observed in any cell cycle stage [37].

*F. carica* latex extract was investigated for its cytotoxic effects on prostate and lung cancer cell lines. Aysin et al. [34] reported that latex extract dose-dependently reduced cell viability in A549 lung cancer cells, and the IC50 value was 1/26 dilution [34]. The same observation was documented by Baohong et al., namely that the alcoholic extract of fig fruit latex in A549 xenograft mice significantly inhibited the tumor growth without inducing obvious damage to the liver or kidney normal mouse tissues [51]. Similarly, in another study, the effects of latex obtained from different fig varieties on PC-3 prostate cancer cell line were evaluated; while viability decreased from 84.7% to 19.1% in Sarı Lop variety, this rate decreased from 80.6% to 2.0% in Black variety. These findings indicate that *F. carica* latex has significant cytotoxic potential against both cancer types [35].

The leaf extract of *F. carica* has been the subject of investigation in the context of skin and laryngeal cancer cell lines. A study demonstrated that *F. carica* leaf extract exhibits cytotoxic effects on melanoma cancer cells (B16F10). Notably, the extract decreased cell viability by 50% at 750 μg/mL after 24 h of incubation and 650 μg/mL after 48 h. Nonetheless, these concentration levels did not result in notable toxicity in normal HEK-293 epithelial cells. Furthermore, findings from the study suggested that the extract induced programmed cell death in melanoma cells via the mitochondrial pathway, leading to an upregulation of *caspase-3*, *caspase-9*, *P53*, and *BAX* gene expression. Notably, at elevated doses (650–750 μg/mL), the expression of apoptosis-associated genes exhibited a substantial increase compared to the control reference gene [46]. Additionally, research by Abdel-Rahman et al. [25] provided evidence of the cytotoxic impact of *F. carica* leaf extract on laryngeal cancer cell lines [25].

In pancreatic cancer models, *F. carica* extract-induced autophagy appears to be regulated via the mTOR/AMPK axis, as suggested by concurrent PARP-1 and LC3A/B upregulation. However, upstream regulators such as Nrf2 and MAPK remain to be investigated [6].

### 3.6. Comparative Summary Across Cancer Types

Across different cancer types, *F. carica* extracts commonly induce mitochondrial-mediated apoptosis through ROS generation, P53 activation, and caspase signaling. Latex and leaf extracts show similar molecular patterns, whereas fruit extracts exert milder and more variable responses. When comparing the extracts, latex generally demonstrates the strongest cytotoxic and pro-apoptotic activity but also presents a greater risk of toxicity in normal cells. Leaf extracts exhibit strong anticancer potential with better selectivity, likely due to their high polyphenolic content (quercetin, luteolin, rutin). Fruit extracts tend to show moderate effects, possibly due to lower concentrations of triterpenes and coumarins. These findings suggest that leaf-based extracts may offer the most favorable therapeutic profile, while latex requires further safety validation.

### 3.7. Contradictions and Gaps

Several studies have reported opposing trends regarding ROS modulation, indicating both antioxidant and pro-oxidant behavior depending on extract composition and dose. This duality highlights the need for standardized extraction methods and comparative mechanistic assays to reconcile the discrepancies observed across studies.

A concise summary of the comparative properties of different *F. carica* extracts is presented in Table 5.

## 4. Safety

The safety of *F. carica* and its extracts remains an important issue in the evaluation of their potential therapeutic applications. In popular medicine for several hundred years, the fruits of *F. carica* have been used for laxative, nose bleeding, or skin problems, e.g., leprosy, or as an emollient, demulcent. Also, it was utilized as an antipyretic, aphrodisiac, lithontriptic, or painkiller. The latex of *F. carica* has been employed as an expectorant, diuretic, and anthelmintic. The leaves have been utilized to treat diabetes, among others [52]. Remarkably, *F. carica* exhibits a diverse array of advantageous effects, including, but not limited to, antioxidant, anti-neurodegenerative, antimicrobial, antiviral, anti-inflammatory, anti-arthritic, antiepileptic, anticonvulsant, anti-hyperlipidemic, anti-angiogenic, antidiabetic, anticancer, and antimutagenic properties [53]. In summary, the multifarious utilization could be underpinned by the anti-inflammatory effects of different plant parts, as demonstrated in this study. Hence, the mentioned indications probably determined the categories of utilization, excluding inadequate use, for example, the consumption of the fruit with diarrhea.

Most in vitro studies have reported significant anticancer activity in different cancer cell lines; nevertheless, in vivo evidence regarding toxicity and safety is rather limited and insufficient. Studies have shown that *F. carica* extracts have a selective affinity for cancer cells while showing minimal toxicity to normal cells. However, this selectivity is largely dependent on factors such as the type of extract, the solvent used, the dosage, and the duration of treatment [52].

The systemic effects of *F. carica* extracts at higher doses in in vivo models have not been adequately investigated, and some reports indicate that certain plant parts—particularly the latex—may be toxic to normal cells at elevated concentrations. Given the scarcity of in vivo data and these toxicity concerns, the review cannot provide definitive information regarding the therapeutic applicability of *F. carica* extracts. Therefore, current findings remain insufficient to guide clinical or systemic use, highlighting the need for well-designed in vivo studies, toxicity assessments, and extract standardization before translation into therapeutic practice can be considered. For example, fig latex extracts exhibited cytotoxic effects on normal fibroblast cells (CCD-45SK) at concentrations above 100 µg/mL [19], whereas leaf extracts showed no significant toxicity up to 2 mg/mL in HUVEC cells [39]. These data suggest a relatively wider therapeutic window for leaf extracts.

Furthermore, while several studies describe antioxidant properties that may contribute to its anticancer effects, others indicate potential pro-oxidant activity that could be detrimental under specific conditions. Toxic manifestations reported at high doses include irritation, oxidative damage, or mitochondrial dysfunction, particularly with latex or essential oil fractions rich in thujones and coumarins. To date, there is no standardized toxicological assessment, and the safe-use limits have not been clearly established [54].

On the other hand, in one patient anaphylactic reaction occurred after ingestion of a fresh fig fruit. In that case, the IgE-dependent allergic mechanism was demonstrated based on the positivity of the prick test with fresh *Ficus 5* extract. However, presumably, weeping fig (*Ficus benjamina*) initiated sensitization in that patient [55]. Furthermore, rarely acute contact urticaria to *F. carica* may occur [56]. Hence, ficin is the major allergen component of *F. carica*, belonging to the cysteine protease family, such as Der p 1 [57].

Furthermore, ficin can activate the coagulation factor X, a key step in the blood clotting cascade [58]. Also, the vitamin K in *F. carica* can interfere with the effectiveness of anticoagulant drugs (blood thinners) [53,59].

Another critical concern is the complete lack of clinical studies evaluating the long-term safety of *F. carica* in humans. Without comprehensive pharmacokinetic and toxicological evaluations, it is difficult to determine the safe dosage range for therapeutic use. In addition, interactions with conventional chemotherapy or other drugs have not yet been investigated, raising concerns about possible side effects [53]. Given these uncertainties, comprehensive in vivo and longitudinal randomized controlled trials are imperative to evaluate *F. carica* extracts’ safety. Standardized toxicity assessments, dose optimization studies, and long-term safety analyses will be critical to determining whether *F. carica* can be safely incorporated into anticancer treatment strategies. Until evidence is available, caution should be exercised when evaluating its use beyond experimental settings.

## 5. Discussion

*F. carica* is a plant with a long history of use in traditional medicine and shows remarkable potential in modern oncological research. Current literature suggests that extracts derived from *F. carica* fruit, leaves, and latex can exhibit anticancer activity in various cancer cell lines, such as pancreas, ovary, stomach, osteosarcoma, lung, prostate, skin, and larynx cancer models. These effects have been associated with mitochondrial-mediated apoptosis, increased ROS production, activation of anti-proliferative mechanisms, induction of autophagy, and, in some cases, initiation of alternative cell death pathways such as ferroptosis. In addition, some studies have shown that compounds isolated from *F. carica* may also play a role in tumor suppression via the immune system. Nonetheless, the mechanisms explaining the pharmacological effects of *F. carica* and the specific bioactive compounds that cause these effects remain largely undefined. A significant limitation of the current findings is that the anticancer effect is largely based on in vitro data. A critical parameter in assessing the clinical applicability of herbal extracts is whether the concentrations used are pharmacologically relevant. Although no universally accepted standard defines precise potency thresholds for crude extracts, the literature commonly refers to approximate ranges to guide interpretation. Generally accepted efficacy thresholds for extracts in the literature are reported as IC50 < 50 µg/mL for strong cytotoxicity, 50–100 µg/mL for moderate activity, and > 200 µg/mL for poor pharmacological applicability. These ranges, however, are not definitive and may vary depending on experimental design and methodological differences among studies [60,61]. While some of the studies included in this review demonstrated efficacy below this range, some examples, particularly latex and polar extracts, appear to produce cytotoxic effects only at very high concentrations [25,33]. This suggests that the observed in vitro effect may have limited translational relevance in terms of in vivo bioavailability and tissue distribution. Significant inconsistencies among studies are due to methodological differences, such as the plant parts from which the extracts were prepared, the solvents used, doses, application times, and the variety of cell lines used in the studies. This heterogeneity complicates the comparative assessment of the anticancer potential of *F. carica*. Also, the use of crude extracts in many studies leaves the true biological source of the anticancer effect unclear. Given the known low oral bioavailability, rapid metabolic transformation, and poor systemic stability of phenolic compounds, the use of extracts as direct therapeutic agents is limited from a pharmacokinetic perspective. Therefore, the use of biodirected fractionation methods to isolate individual bioactive compounds and define the pharmacodynamic mechanisms specific to these molecules appears to be a more ra-tional approach. Indeed, some isolated compounds appear to have significantly lower IC50 values compared to crude extracts. Regarding safety, although many studies indicate that *F. carica* extracts selectively target cancer cells and cause minimal toxicity to normal cells, these results are generally short-term and confined to cell culture. It has been reported that some plant parts, especially latex, can show toxicity in normal cells at high concentrations. Also, contrary to some findings, limited data suggest that F. carica may promote tumor development under certain conditions, highlighting the condition-dependent effects of the plant. Therefore, the safety profile should be examined from a broad perspective, not only through toxicity assessments but also by identifying potential tumor-promoting effects. Moreover, in addition to antioxidant effects, the potential for pro-oxidant activity may also pose a risk under certain conditions. Currently, there is insufficient pharmacokinetic, toxicological, or clinical evidence to evaluate the long-term safety of *F. carica* in humans.

## 6. Conclusions

In conclusion, although *F. carica* demonstrates promising anticancer potential against various types of cancer, the bioactive compounds mediating these effects and their underlying mechanism remain inadequately characterized. Before clinical application, comprehensive in vivo studies and well-designed controlled clinical trials utilizing standardized extracts are essential to rigorously evaluate both the efficacy and safety profile of *F. carica*. For example, in preclinical small animal studies, a careful choice of parts (excluding leaves) or purification of components, or an absence genetic version of *F. carica* will be needed to avert anticoagulant effects of coumarin as well as liver- and neurotoxic effects of thujones. Such studies are critical to determine whether *F. carica* can be incorporated into anticancer treatment strategies on a scientific basis. Future research should elucidate the upstream signaling regulators, such as Nrf2, MAPK, and mTOR, that may integrate oxidative stress responses and autophagy pathways induced by *F. carica* compounds.

Here we need to highlight that potential allergic and anticoagulant effects, as well as blood sugar level-decreasing effects of *F. carica*, indicate precautions before treatment of potentially sensitive patients with its extracts or even the first consumption of the fruits.

## Figures and Tables

**Table 1:** Phytochemical content of *F. carica* according to its parts [14,16,23].

Compound Type	Fruits	Leaves	Latex
Flavanols	Quercetin, catechin, rutin, epicatechin, gallocatechin, kaempferol	Epicatechin, catechin, taxifolin, kaempferol, rutin, quercetin	-
Flavones	Chrysin, apigenin, galangin	Isoorientin, orientin, apigenin, luteolin, vitexin, biochanin A/B,	-
Flavanones	Pinostrobin, pineocembrin	Hesperidin, naringenin	-
Anthocyanins	(Epi)catechin-(4 → 8) cyanidin-3,5-diglucoside, cyanidin-3 glucoside/rutinoside	-	-
Coumarins	Scopoletin, bergapten, psoralen, umbelliferone	Psoralen, umbelliferone	-
Monoterpenes	Menthol, α-pinene, β-pinene, limonene, inasole	Menthol, limonene	Limonene, α-thujene, α-pinene, β-pinene
Sesquiterpenes	α-Caryophyllene, β-copaene, α-zingiberene, β-caryophyllene, (E)-β-bergamotene, β-thujone	α-Cubebene, α-zingiberene, β-caryophyllene, α-humulene, β-eudesmol, α-bourbonene	-
Triterpenes/Tetraterpenes	β-carotene, zeaxanthin, α-carotene, β-cryptoxanthin, lutein	α-Taraxasterol acetate, bauerenol, lupeol acetate	Lupeol, β-amyrin, α-amyrin, lanosterol
Hydroxybenzoic Acids	Gallic acid	Gallic acid, syringic acid, protocatechuic acid	-
Hydroxycinnamic Acids	p-Coumaric, ferulic, chlorogenic, cinnamic	p-Coumaric, ferulic, chlorogenic	-

**Table 2:** Phytosterol and organic acid content of *F. carica* according to its parts [14,23].

Compound Type	Fruits	Leaves	Latex
Phytosterols	β-Sitosterol, stigmasterol, campesterol	β-Sitosterol	β-Sitosterol
Organic Acids	-	Citric, oxalic, fumaric, malic, and quinic acid	-

**Table 3:** The potential anticancer effects and underlying mechanisms of *F. carica* based on in vitro research.

Type of Cancer	Cell Line	Part of *F. carica*	Dosage and Exposure Time	(+) Effects	No Effect/ (−) Effects	Mechanisms	References
Breast	AMJ13	Fruit extract (n-hexane) 2-benzhydrylsulfinyl-N-hydroxyacetamide-Na isolation (methanol solvent)	100, 50, 25, 12,5, 6,25 μg/mL 72 h.	↓ cell proliferation, ↓ cell viability, ↑ apoptosis	Not specified	↓ MMP, ↓ BCL-2, ↑ BAX, ↑ P53, ↑ caspase-8 ↑ caspase-9	[24]
Breast	MCF7	Leaf extract (ethanol solvent)	5000, 2500, 1250, 625, 312.5, and 156 µg/mL 48 h.	↓ cell viability, ↑ peripheral blood mononuclear cells	Not specified	↑ ROS	[25]
Breast	MDA-MB-231	Latex extract (no specified)	%0.1, %0.25, %0.5 ve %1 24, 48, and 72 h	↑ apoptosis, ↓ cell proliferation, ↓ cell viability, ↓ cell migration, ↑ cytotoxicity and genotoxicity	Not specified	↓ ERK, ↓ CREB, ↓ AKT, ↓ GSK-3α/β, ↓ AMPK ↑ DNA damage	[28]
Breast	MCF7	Latex extract (ethanol solvent)	5 -400 µg/mL 4 h.	↑ cytotoxicity ↓ cell viability	healthy CCD45-SK cells cytotoxicity ↑	Not specified	[19]
Breast	MDA-MB-231	Leaf extract nanoparticles	50 µ M 24 h.	↑ cytotoxicity ↑ anti-tumor activity	Not specified	Not specified	[29]
Breast	MCF7	Fruit extract (methanol solvent)	500 µg mL^−1^ 24–72 h.	↑ cytotoxicity ↑ apoptosis	Not specified	Not specified	[30]
Breast	MCF7	Leaf extract (ethanol solvent)	8 mg/mL Not specified	↑ cytotoxicity	Not specified	Not specified	[31]
Breast	MCF7 MDA-MB231 MDA-MB436	Leaf extract (ethanol solvent)	From 6.25 to 300 μg/mL 72 h.	↑ cytotoxicity ↓ cell viability	Not specified	↑ Caspase-3	[32]
Breast	MCF7	Wood bark extract (asetone solvent)	From 5 to 100 µg/mL 4 h.	↑ cytotoxicity ↓ cell viability	>100 µg/mL healthy CCD45-SK cells viability ↓	Not specified	[33]
Breast	MCF7 MDA-MB231	Latex extract (Not specified)	1/1500–1/12 48 h.	↑ cytotoxicity ↓ cell viability	Not specified	Not specified	[34]
Colon	CaCo2	Leaf extract (ethanol solvent)	5000, 2500, 1250, 625, 312.5, and 156 µg/mL 48 h.	↓ cell viability ↑ peripheral blood mononuclear cells	Not specified	↑ ROS	[25]
Colon	HT29	Latex extract (pure water phosphate-buffered saline solvent)	10, 20, 25, 30, 40, 50, 75, 100 μg/mL 24, 48, and 72 h	↑ cytotoxicity ↑ apoptosis	↔ cytotoxicity (10 μg/mL) ↔ apoptosis (40 μg/mL 24 h.)	↑ Caspase-3/7	[35]
Colon	HCT116	Latex extract (ethanol solvent)	5 -400 µg/mL 4 h.	↑ cytotoxicity ↓ cell viability	healthy CCD45-SK cells cytotoxicity ↑	Not specified	[19]
Colon	C26	Leaf extract (petroleum ether solvent) Bergapten isolation Cubic nanoparticles	10 µM and 100 µM 48 snd 72 h. 22 days.	↑ cytotoxicity ↓ cell viability ↓ tumor growth	Not specified	Inhibit P-glycoprotein	[36]
Colon	CaCo2	Leaf extract (ethanol solvent)	8 mg/mL Not specified	Not found	↔ cytotoxicity	Not specified	[31]
Colon	HT29	Fruit extract (n-hexane, dichloromethane, methanol, ethanol, Water)	1/10–1/100,000 (3330 mg/mL stock solution) 24–72 h.	↑ cytotoxicity ↑ apoptosis ↑ Cell Cycle Arrest ↓ cell viability ↓ cell proliferation	Not specified	Not specified	[37]
Colon	CaCo2	Latex (Cellulose acetate/polyethylene oxide nanofiber)	1, 5, 10, 15, 30, 60 ve 120 μg/mL 24 h.	↑ cytotoxicity ↑ apoptosis	↓ TP53, ↓ TNF-α, ↑ Bcl2 ↓ P21(10 μg/mL)	↑ P21	[17]
Colon	HT116	Wood bark extract (asetone solvent)	From 5 to 100 µg/mL 4 h.	↑ cytotoxicity ↓ cell viability	>100 µg/mL healthy CCD45-SK cells viability ↓	Not specified	[33]
Colon	HT29	Latex extract (methanol solvent)	0,5–2,5 mg/mL 48 h.	↓ cell viability ↑ anti-colonization	Not specified	Not specified	[38]
Liver	HepG2 NIH3T3	Latex extract (ethyl acetate,n-hexane, chloroform, and methanol solvents)	0.6, 1.25, 2.5, 5 and 10 mg/mL 24, 48, and 72 h.	↑ cytotoxic effect ((n-hexane, chloroform, ethyl acetate form) ↑ apoptosis (chloroform)	↑ growth stimulation activity(methanol form) ↔ cell death	Not specified	[26]
Liver	HepG2	Leaf extract (ethanol solvent)	5000, 2500, 1250, 625, 312.5, and 156 µg/mL 48 h.	↓ cell viability ↑ peripheral blood mononuclear cells	↔ cell viability (156 µg/mL)	↑ ROS	[25]
Liver	HepG2	Leaf extract (asetone solvent)	0.125, 0.25, 0.5, 1, 2, and 4 mg/mL 24, 48, and 72 h	↑ cytotoxicity ↑ apoptosis ↓ cell proliferation ↑ cell cycle arrest ↑ anti-colonization	↑ CDK-10, ↓ TP53	↑ ROS, ↓ MMP, ↓ Bcl-2, ↓ CDK1/5/9, ↓ DNA synthesis	[39]
Liver	HepG2	Latex extract (ethanol solvent)	5 −400 µg/mL 4 h.	↑ cytotoxicity ↓ cell viability	healthy CCD45-SK cells cytotoxicity ↑	Not specified	[19]
Liver	HepG2	Fruit extract essential oils isolation Water and n-hexane solvent)	Not specified	↑ Cell Cycle Arrest (water form) ↑ apoptosis ↓ cell viability	↔Cell Cycle Arrest (n-hexane form) ↓ ROS	Not specified	[40]
Liver	HepG2	Fruit extract (methanol solvent)	500 µg mL^−1^ 24–72 h.	↑ cytotoxicity ↑ apoptosis	Not specified	Not specified	[30]
Liver	HepG2	Latex (Cellulose acetate/polyethylene oxide nanofiber)	1, 5, 10, 15, 30, 60 ve 120 μg/mL 24 h.	↑ cytotoxicity ↑ apoptosis	↓ TP53, ↓ TNF-α, ↑ Bcl2 ↓ P21(10 μg/mL)	↑ P21	[17]
Liver	HepG2	Wood bark extract (asetone solvent)	From 5 to 100 µg/mL 4 h.	↔ cell viability	>100 µg/mL healthy CCD45-SK cells viability ↓	Not specified	[33]
Cervical	HeLa	Fruit extract (ethanol solvent)	Not specified	Not found	↔ cytotoxic effect	Not specified	[27]
Cervical	HeLa HPV18+ CaSki HPV16+	Latex extract (DMSO solvent)	5, 10, 50, 100, and 200 μg/mL 72 h.	↑ apoptosis ↑ cytotoxicity ↓ Cell growth ↑ Cell Cycle Arrest	Not specified	Nonsense-Mediated Decay pathway (↑ RPS27A, RNF111, and RPS6) Cell Cycle pathway (↓ PCNA, POLD3, PRIM1, and ORC2) ↑ TP53, ↓ E6 and E7 oncoproteins ↓ CDK4, CDK1 and MAPK1	[41]
Cervical	HeLa(HPV18+) CaSki(HPV16+) C33A(HPV−)	Latex extract (DMSO solvent)	5, 10, 50, 100, and 200 μg/mL 72 h.	↑ cytotoxicity ↑ Cell Cycle Arrest ↓ Cell growth ↑ apoptosis	Not specified	Degradation and Antigen Presentation via the Ubiquitin-Proteasome and MHC-I Pathways (↑ RPS27A, RNF111, CUL5, FBXO4, FBXL4, and CALR) ↓ E5 oncoprotein	[42]
Cervical	HeLa	Latex extract (methanol solvent)	0.5–2.5 mg/mL 48 h.	↓ cell viability ↑ anti-colonization	Not specified	Not specified	[38]
Pancreas	Panc1 QGP1	Fruit extract (ethanol solvent)	150, 300, 600 μg/mL and 1.2 mg/mL	↑ cytotoxicity ↑ apoptosis ↑ cell death ↓ cell viability ↓ cell migration, ↓ metastasis and invasion ↑ anti-colonization ↑ cell senescence and autophagy	↔ Caspase 3, 7, 8 and 9	↓ PARP-1, ↓ N –kadherin, ↑ E-kadherin, ↑ ROS, ↓ Atg-5, ↓ Beclin-1 ve ↓ p62, ↑ LC3A/B, ↓ MMP, ↑ lipid peroxidation	[43]
Pancreas	Panc1 AsPC1	Fruit extract (ethyl acetate solvent)	0.1, 0.5, and 1 mg/mL 24 h.	↑ cytotoxicity	↔ cell proliferation ↑ cell migration	Not specified	[44]
Ovarian	SKOV3	Fruit extract (n-hexane) 2-benzhydrylsulfinyl-N-hydroxyacetamide-Na isolation (methanol solvent)	100, 50, 25, 12.5, 6.25 μg/mL 72 h.	↓ cell proliferation ↓ cell viability ↑ apoptosis	Not specified	↓ MMP, ↓ BCL-2, ↑ BAX, ↑ P53, ↑ caspase-8 ↑ caspase-9	[24]
Ovarian	SKOV3	Fruit extract (n-hexane) 2-(Benzhydryl sulfinyl)-N-sec-butylacetamide) isolation (methanol solvent)	25 µg/mL 60 min. 500 µg/kg	↑ bone marrow-derived macrophages phagocytic activity ↑ anti-tumor activity	Not specified	↑ Fcγ receptor expression ↑ ROS	[45]
Gastric	AGS	Fruit extract (methanol solvent)	20, 40, 60, 80, 100 mg/mL 24 h.	↑ cytotoxicity ↓ cell viability ↑ apoptosis	Not specified	↑ ROS, ↓ Glutathione, ↓ MMP, ↑ DNA damage	[18]
Gastric	AGS	Leaf extract (ethanol solvent)	8 mg/mL Not specified	↑ cytotoxicity	Not specified	Not specified	[31]
Osteosarcoma	MG63	Fruit extract (n-hexane, dichloromethane, methanol, ethanol, Water)	1/10–1/100,000 (3330 mg/mL stock solution) 24–72 h.	↑ cytotoxicity ↑ apoptosis ↓ cell viability ↓ cell proliferation	↔ Cell Cycle Arrest	Not specified	[37]
Lung	A549	Latex extract (Not specified)	1/1500–1/12 48 h.	↑ cytotoxicity ↓ cell viability	Not specified	Not specified	[34]
Prostate	PC3	Latex extract (pure water phosphate-buffered saline solvent)	10, 20, 25, 30, 40, 50, 75, 100 μg/mL 24, 48, and 72 h	↑ cytotoxicity	Not specified	↑ Caspase-3/7	[35]
Skin	B16F10	Leaf extract (methanol solvent)	150, 250, 350, 450, 550, 650, 750 ve 850 μg / mL 24 and 48 h.	↑ cytotoxicity ↑ apoptosis	Not specified	↑ p53, ↑ Bax, ↑ Caspase 3 and 9	[46]
Laryngeal	Hep2	Leaf extract (ethanol solvent)	5000, 2500, 1250, 625, 312.5, and 156 µg/mL 48 h.	↓ cell viability ↑ peripheral blood mononuclear cells	↔ cell viability (156 µg/mL)	↑ ROS	[25]

**AGS**: Adenocarcinoma Gastric Cell Line, **AKT**: Protein Kinase B, **AMPK**: AMP-activated Protein Kinase, **Atg-5**: Autophagy-Related 5, **Bax**: Bcl-2-associated X Protein, **Bcl-2**: B-cell Lymphoma 2, **CaCo2**: Human Colorectal Adenocarcinoma Cell Line, **CALR**: Calreticulin, **CaSki**: Cervical Cancer Cell Line (HPV16+), **CDK**: Cyclin-Dependent Kinase, **CREB**: cAMP Response Element-Binding Protein, **CUL5**: Cullin 5, **DNA**: Deoxyribonucleic Acid, **DMSO**: Dimethyl Sulfoxide, **E-cadherin**: Epithelial Cadherin, **E5/E6/E7**: Human Papillomavirus (HPV) Oncoproteins, **ERK**: Extracellular Signal-Regulated Kinase, **FBXL4**: F-box and Leucine-Rich Repeat Protein 4, **FBXO4**: F-box Only Protein 4, **Fcγ receptor**: Fc Gamma Receptor, **GSK-3α/β**: Glycogen Synthase Kinase 3 Alpha/Beta, **HeLa**: Human Cervical Cancer Cell Line, **Hep2**: Human Laryngeal Carcinoma Cell Line, **HepG2**: Human Hepatocellular Carcinoma Cell Line, **HPV**: Human Papillomavirus, **HT29**: Human Colorectal Adenocarcinoma Cell Line, **LC3A/B**: Microtubule-associated Protein 1A/1B-light Chain 3A/B, **MAPK1**: Mitogen-Activated Protein Kinase 1, **MCF7**: Human Breast Adenocarcinoma Cell Line, **MDA-MB-231/MDA-MB-436**: Triple-negative Breast Cancer Cell Lines, **MHC-I**: Major Histocompatibility Complex Class I, **MG63**: Human Osteosarcoma Cell Line, **MMP**: Mitochondrial Membrane Potential, **NIH3T3**: Mouse Embryonic Fibroblast Cell Line, **N-cadherin**: Neural Cadherin, **ORC2**: Origin Recognition Complex Subunit 2, **P21**: Cyclin-Dependent Kinase Inhibitor 1A, **PARP-1**: Poly(ADP-ribose) Polymerase 1, **PC3**: Human Prostate Cancer Cell Line, **PCNA**: Proliferating Cell Nuclear Antigen, **POLD3**: DNA Polymerase Delta 3, **PRIM1**: DNA Primase Subunit 1, **QGP1**: Human Pancreatic Neuroendocrine Tumor Cell Line, **RNF111**: Ring Finger Protein 111, **ROS**: Reactive Oxygen Species, **RPS27A**: Ribosomal Protein S27a, **SEC**: Secundum, **SKOV3**: Human Ovarian Cancer Cell Line, **TP53**: Tumor Protein p53, **TNF-α**: Tumor Necrosis Factor Alpha, **Ubiquitin-Proteasome**: Protein Degradation Pathway.

**Table 4:** The potential anticancer effects and underlying mechanisms of F. carica based on in vivo research.

Animal Model	Plant Part/Extract	Dose & Duration	Evaluated Toxicity Parameters	Main Findings	References
Balb/c mice	Fruit extract (n-hexane) 2-benzhydrylsulfi-nyl-N-hydroxyacetamide-Na isolation	1 mg/kg (intraperitoneal) 4 weeks	The liver, kidney, lung, spleen histopathology and liver enzymes GOT, GPT, and ALP.	No significant changes and toxicity.	[24]
Syrian mice	Latex extract (chloroform, and methanol solvents)	1, 2 and 3 g/kg (intraperitoneal) 14 days	abdominal organs and liver enzymes AST, ALT, and ALP	3 deaths in 3 g/kg; no death in 1 and 2 g/kg. No significant changes and toxicity in 1 and 2 g/kg; significant changes and toxicity in 3 g/kg.	[26]
BALB/c mice	Fruit extract (ethanol sol-vent)	200 mg/kg) once a day (gavage) 7 days.	tumor growth and cytotoxicity on normal pancreatic cells	Tumor volume and tumor weight significantly lower than in the control group. No significant toxicity in normal pancreatic cells.	[43]
Mice	Leaf extract (Bergapten isolation Cubic nanoparticles)	10 µM–100 µM (intravenous) 22 days.	Anti-tumor activity	Inhibited tumor growth.	[36]
C57/BL6 mice	Fruit extract (n-hexane) 2-(Benzhydryl sulfi-nyl)-N-sec-butylacetamide) isolation	500 µg/kg (intraperitoneal)	Tumoricidal activity	Enhanced the functional activities of bone marrow-derived macrophages against tumor cells.	[45]
Wistar rats	Latex extract	0.5 mL (intratumorally injected) 4 weeks	tumor growth	Significant reduction in the tumor volume.	[47]
Albino Wistar rats	Morin	50 mg/kg/day (intragastric intubation) 30 days.	tumor multiplicity BAX/BCL2 ratio anti-inflammatory effects	Significantly reduced tumor multiplicity. Modulating BAX/BCL2 ratio. Regulating cytokines and eicosanoid. Attenuated NF-κB signaling in colon tumors	[48]

GOT: Glutamic Oxaloacetic Transaminase, GPT: Glutamic Pyruvic Transaminase, ALP: Alkaline Phosphatase, AST: Aspartat Aminotransferaz, ALT: Alanin Aminotransferaz.

**Table 5:** Summary of the comparative properties of different *F. carica* extracts.

Extract Type	Typical Mechanisms	Efficacy	Toxicity Profile	References
Latex	ROS ↑, Caspase ↑, p53 ↑	Strong	Moderate–High	[19,26,28,41]
Leaf	ROS ↑/↓, Bcl-2 ↓, Caspase ↑	Strong	Low	[25,32,39]
Fruit	Moderate apoptosis, variable ROS	Moderate	Low–Moderate	[18,24,43]
Bark	Limited data	Weak	Low	[33]

## Data Availability

No new data were created or analyzed in this study. Data sharing is not applicable to this article.
